# Effect of Cognitive Decline on Mandibular Movement during Mastication in Nursing Home Residents

**DOI:** 10.3390/jcm13206040

**Published:** 2024-10-10

**Authors:** Enri Nakayama, Haruka Tohara, Masanori Kimura, Iki Koide, Kimiko Abe, Kazumichi Yonenaga

**Affiliations:** 1 The Department of Dysphagia Rehabilitation, Nihon University School of Dentistry, Tokyo 1018310, Japan; crycrycry@outlook.jp (I.K.); abe.kimiko@nihon-u.ac.jp (K.A.); yonenaga.kazumichi@nihon-u.ac.jp (K.Y.); 2 Dysphagia Rehabilitation, Department of Gerontology and Gerodontology, Graduate School of Medical and Dental Sciences, Institute of Science Tokyo, Tokyo 1138510, Japan; harukatohara@hotmail.com; 3 Department of Dysphagia Rehabilitation, Division of Oral Pathogenesis and Disease Control, Asahi University School of Dentistry, Gifu 5010296, Japan; melancholy_of_saga@yahoo.co.jp

**Keywords:** mastication, eating, cognitive function, geriatrics, long-term care facility

## Abstract

**Background**: Many studies have reported on the relationship between cognitive and masticatory functions. However, it remains unclear how the mandibular movements change during chewing in facility residents as dementia progresses. This study aimed to investigate the relationship between a kinematic analysis of mandibular movement during mastication and cognitive function in facility residents. **Methods:** Sixty-three participants were included from two long-term care facilities. The primary outcome variable was the kinematic data of mandibular movement during mastication. The participants chewed rice crackers, and their faces were recorded during this motion. The partial correlation coefficient between kinematic data and cognitive function was calculated. Furthermore, group comparisons were performed after dividing the participants into three groups based on their cognitive function. **Results:** Circular motion frequency was significantly correlated with the ABC dementia scale, even after adjusting for the appendicular skeletal muscle index, Eichner index, and short-form mini-nutritional assessment. The cycle and circular motion frequencies were markedly lower in the severe dementia group than in the mild dementia group. **Conclusions:** With declining cognitive function, mandibular movements during mastication decrease in circular motion and increase in linear motion. Additionally, our results suggested that residents with severe cognitive impairment had more linear mandibular motions during mastication than those with mild cognitive impairment. This may make it more difficult for residents with cognitive decline to ingest normal solid foods.

## 1. Introduction

A satisfactory diet is crucial for older people in long-term care facilities [1], and its continuity is essential for maintaining their quality of life [2]. Unfortunately, many care recipients face challenges in consuming a satisfactory normal diet, as their masticatory function declines with an increasing level of required care [3].

Masticatory movements, both voluntarily controlled and semiautomatic, are rhythmically repeated movements. These are governed by the cerebrum and masticatory central pattern generator (CPG) in the brainstem [4]. Additionally, they are adjusted based on food properties, such as hardness and viscoelasticity, sensed by sensory receptors distributed in the periodontal ligament, temporomandibular joints, and spindles of the masticatory muscles [5,6]. Therefore, diseases that cause damage to areas related to mastication, such as cerebrovascular diseases [7], neuromuscular diseases [8], and dementia [9], can impair masticatory movements. In addition, a decrease in the number of remaining teeth [10] and occlusal supports [11] and weakened masticatory and tongue muscles resulting from sarcopenia [12] may diminish masticatory efficiency. As described above, elderly individuals who require care face various risks of deteriorating masticatory function. In particular, there are many residents in facilities who suffer from dementia; therefore, the negative effects of dementia on masticatory function are of great concern.

Dementia constituted 23.16% of the causes of long-term care in Japan in 2022, making it the most common cause of illness [13]. Additionally, the number of patients with dementia in Japan is increasing annually and expected to reach approximately 7 million by 2025 [14]. Several recent studies have reported on the relationship between cognitive and masticatory functions [15]. However, no reports have examined the correlation between the kinematic analysis results of mandibular movements during mastication and cognitive function in elderly individuals requiring care. In recent years, the Saku-Saku test has been utilized to evaluate masticatory function by observing the mandibular movement during mastication [16]. It classifies individuals into good and poor mandibular rotation according to mandibular movement while eating rice crackers. Good mandibular rotation is defined as when the mandible opens centrally or slightly toward the masticatory side and closes laterally in the opening path. Meanwhile, poor mandibular rotation is defined as a simple linear movement opening and closing along a similar path. This method has been proven to have high reliability between examiners and reporters and is useful for evaluating masticatory function in patients with dysphagia. In a previous study, we used the Saku-Saku test to compare mandibular movements during mastication between people consuming a normal diet and those with a dysphagic diet in a nursing home. Significant differences were observed between the normal and dysphagia diet groups in terms of masticatory time, cycle frequency, total change, number of linear motions, and circular motion frequency. Additionally, analyzing the frequency of circular motion has been suggested as a screening test to identify individuals who require dysphagia diets [17]. This study found that residents who could not eat a normal diet tended to have lower circular motion frequencies though we did not verify the factors contributing to this. Many of the residents who required dysphagia diets had dementia; hence, we hypothesized that a decrease in the frequency of circular motion might be associated with the progression of dementia.

The purpose of this study was to clarify the relationship between a kinematic analysis of mandibular movement during mastication and cognitive function in facility residents. This study may clarify the factors that may make it difficult for residents of nursing homes to ingest normal solid foods. Furthermore, the results of this study may suggest when residents with dementia are no longer able to perform the mandibular movements appropriate for normal solid food intake.

## 2. Materials and Methods

### 2.1. Participants

This cross-sectional study was conducted at two facilities in Japan between 2020 and 2021. The conditions for participation were as follows: residents orally ingesting solid foods and consenting to this study. Exclusion criteria were as follows: (1) inability to fulfill simple requests due to cognitive decline; (2) inability to eat while sitting on a chair; (3) too weak to withstand examination; (4) no occlusal contact with molars (including dentures); and (5) a history of oral cancer, dental disorders causing painful mastication (including temporomandibular joint disorders), or defective dentures. The sample size was calculated using G * Power (ver. 3.1.9.4; Universität Kiel, Kiel, Germany) with an effect size f of 0.4, α of 0.05, and power (1 − β) of 0.8; the results revealed that a minimum sample size of 66 participants was necessitated. Nurses in the facilities selected the participants, and all participants or their families received a verbal and written explanation of this study and provided written informed consent. This study was approved by the Institutional Review Board (approval no. EP19D015-1) and was conducted in accordance with the Declaration of Helsinki.

### 2.2. Outcome Measures

Color stickers (5 mm in diameter) were attached to the most protruding parts of the cheekbones, apex of the nose, and tip of the participant’s mandible. A throat microphone (Inkou mike; NZ-210CjK, NANZU, Shizuoka, Japan) was affixed approximately 2 cm outside the laryngeal prominence of the thyroid cartilage. The microphone recorded swallowing sounds due to the passage of the bolus through the pharynx. Participants, seated in wheelchairs or armchairs, wearing dentures if necessary, first took a sip of water with viscosity adjusted as usual. They were then given one-third of a baby rice cracker (Hai Hain^®^ Baby rice cracker, Kameda Seika Co., Ltd., Niigata, Japan) for a trial exercise, becoming muddy after chewing. Observations during the trial exercise led to the exclusion of participants judged to be at high risk of aspiration or choking in subsequent procedures. Subsequently, after another sip of water, they were given approximately 2 g of rice cracker (Happy Turn^®^ soft rice cracker, Kameda Seika Co., Ltd., Niigata, Japan) split in half [16], and they were instructed to keep their faces still while looking at the camera and chewing. An iPad (iPad Pro, Apple Inc., Cupertino, CA, USA) held directly in front of the participant’s face recorded mandibular movements along with corresponding swallowing sounds from the throat microphone [17]. In order to minimize stress for the participants, the test was conducted in a place where they spend their daily lives, and the nursing staff at the facility were asked to give instructions and put the foods in their mouths. However, participants who moved their faces excessively or spoke during mastication were not suitable for measurement, so they were photographed up to two times again, and participants who did not improve after this were excluded.

Recorded images were analyzed by a trained examiner using an image analysis software (DIPP-Motion PRO 2D Ver1.25; DITECT Corporation, Tokyo, Japan). Among the images of participants chewing the rice cracker, the masticatory movement from the first crushing sound to the first swallowing sound was analyzed. The *x*-axis was the line passing through stickers on the left and right cheekbones, and the *y*-axis was the line through the center of the sticker on the apex of the nose, perpendicular to the *x*-axis. Subsequently, the movement of the sticker on the tip of the lower jaw was measured. The period from the start of mandibular descent to the return to the original position was defined as one cycle. Measurements included the time from the start of mastication to swallowing (masticatory time), total number of cycles (number of cycles), cycle frequency (number of cycles/mastication time), total amount of change, average speed, maximum speed, and speed coefficient of variation. Furthermore, the number of times classified into the following types for each cycle according to the mandibular locus was counted. Circular motion was defined as when the mandible opened centrally or slightly towards the chewing side and closed lateral to the opening path. Simple linear movement with opening and closing along a similar path was defined as linear motion. The number of motions was counted, and the circular motion frequency (number of circular motions/total number of cycles) was calculated. A dentist specializing in dysphagia performed these counts using a slow-motion replay of recorded footage displaying chin marker trajectories. This measurement method has high intra- and inter-rater reliabilities [17].

### 2.3. Other Measurements

Nurses and dietitians in the long-term care facilities surveyed the following items immediately prior to the survey: age, sex, history of cerebrovascular disease, activities of daily living (ADLs), body mass index (BMI), and nutritional status. ADLs were assessed using the Government-Certified Disability Index (GCDI) [18]. Nutritional status was assessed by dietitians using the short-form mini-nutritional assessment (MNA-SF) [19]. The appendicular skeletal muscle index (ASMI) was measured using bioelectrical impedance analysis (Seca 525; Seca GmbH & Co., KG, Hamburg, Germany). Calf circumference (CC) was measured on the left foot or the non-paralyzed side. ASMI and CC data were measured by physiotherapists within 2 weeks of the survey date. The dietary form was evaluated on a 3-level scale: normal diet, slightly soft diet, finely minced soft diet covered with a thick sauce or mousse diet. The physiotherapists assessed cognitive function using the ABC dementia scale (ABC-DS) [20]. The ABC-DS is easy to use even for medical staff who are not familiar with assessing cognitive function, as the assessment form is accompanied by easy-to-understand illustrations, and it has excellent validity and reliability. In addition, the ABC-DS protocol does not require the assessor to judge the severity of a patient’s dementia before assessment. The ABC-DS consists of three domains as follows: Domain A, reflecting ADL function; Domain B, reflecting behavioral and psychological symptoms of dementia (BPSD); and Domain C, reflecting cognitive function. The total score ranges from 101 to 117 for normal or suspected dementia, 86 to 100 for mild, 71 to 85 for moderate, and 13 to 70 for severe dementia [21]. On the day of the survey, a dentist collected data on the state of tooth defects, evaluated using the Eichner index [22].

### 2.4. Statistical Analysis

To examine the relationship between kinematic outcomes and other measurements, Spearman’s rank correlation coefficient was used. Additionally, we examined the correlation coefficient between kinematic results and cognitive function after adjusting for related factors. As related factors, we applied muscle mass (ASMI) and occlusal support area (Eichner index), which have been reported to have a direct influence on mastication in previous studies, and items that were found to be related in our survey. Participants were divided into three groups based on the total ABC-DS score: normal or suspected dementia, mild dementia (mild), moderate dementia (moderate), and severe dementia (severe). A one-way analysis of variance and Tukey’s honestly significant difference test between the groups were performed. The level of significance was set at *p* < 0.05. All statistical analyses were performed using SPSS (IBM SPSS Statistics for Windows Version 28.0; IBM Japan Ltd., Tokyo, Japan).

## 3. Results

### 3.1. The Characteristics of the Participants

**Figure 1:**
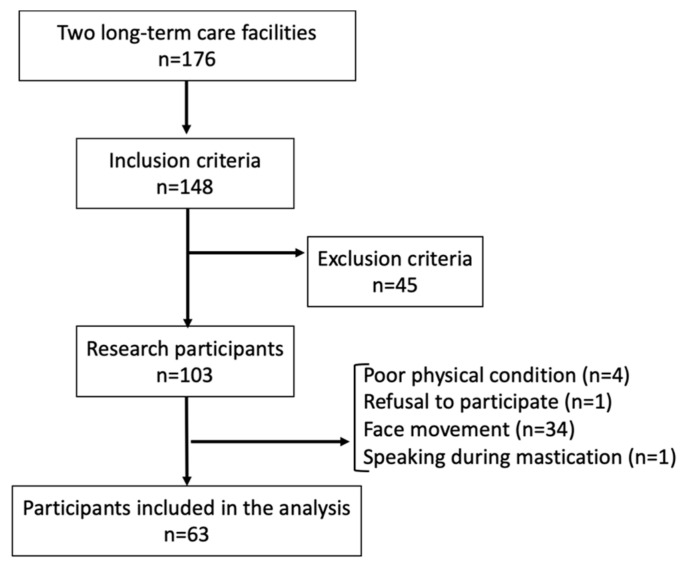
A flow chart of the participants.

There were 176 residents in both facilities, and 148 participants met the inclusion criteria. A total of 45 participants were excluded, and 103 underwent measurements. However, 40 individuals were excluded for various reasons on the day of measurement. Therefore, an analysis was performed using the recorded images of the 63 participants (Figure 1).

The average age of the participants was 88.1 years, with 15 male and 48 female individuals. Additionally, 20 patients had a history of cerebrovascular disease, and 2 had a history of Parkinson’s disease. Besides dementia, none of the participants had a history of any diseases directly affecting their swallowing function. The mean BMI was 21.03 kg/m^2^, and the mean ASMI was 6.12 kg/m^2^. Seven participants were malnourished, as assessed by the MNA-SF. Based on the ABC-DS total score, 21, 18, and 24 participants were classified into the normal, moderate, and severe groups, respectively. The participants’ characteristics are summarized in Table 1.

### 3.2. Correlation Coefficient with Kinematic Results

After examining the correlation between the kinematic results and measurement items, no items were found to be significantly related to masticatory time, number of cycles, total change amount, average speed, or maximum speed. Meanwhile, the cycle frequency was significantly associated with ABC-DS scores. Additionally, the MNA-SF, ASMI, and ABC-DS scores were all significantly related to the number of circular motions and circular motion frequency (Table 2).

The relationship between the kinematic results and the ABC-DS was investigated using partial correlation coefficients adjusted for the MNA-SF, ASMI, and Eichner index. The number of cycle motions and circular motion frequency were significantly associated with the total ABC-DS score. However, Domain B showed no correlation with any of the items (Table 3).

### 3.3. Relationship between Kinematic Results and Dementia Severity

**Figure 2:**
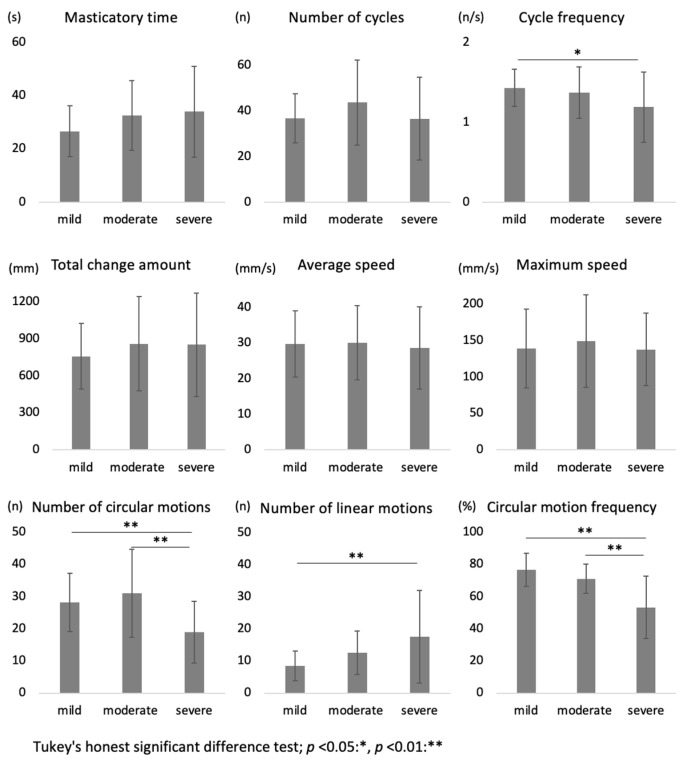
Relationship between kinematic results and dementia severity. All analyses were performed using Tukey’s honestly significant difference test.

Participants were classified into mild, moderate, and severe groups based on the ABC-DS total score. The mean ages were 91.2, 86.8, and 86.2 years, respectively. There was no significant difference in age between the groups. The cycle frequency, number of circular motions, and circular motion frequency were significantly lower, and the number of linear motions was significantly higher in the severe group than in the mild group. Furthermore, upon comparing the moderate and severe groups, significant differences were observed in the number of circular motions and circular motion frequency. However, no significant differences were observed between the mild and moderate groups (Figure 2).

## 4. Discussion

Our previous study reported that the circular motion frequency was significantly reduced in institutional residents who could not consume a normal diet. However, we did not verify the factors contributing to this [17]. In this study, we obtained results suggesting that the masticatory movements of facility residents are influenced by cognitive function.

Many studies have reported that a decline in cognitive function is associated with a decline in masticatory ability. However, most studies reported to date have measured masticatory efficiency using gum, gummy jelly, and foods of different hardness or estimated masticatory ability using questionnaires regarding intake status [15]. Therefore, the mandibular movements during mastication in patients with dementia remain unclear. This research was conducted using video recordings of the mastication of snack food from the start of chewing until swallowing and then analyzing the video recordings. Unlike methods using gum or gummy jelly, this method does not necessitate spitting the test foods; hence, more natural masticatory movements can be observed. In this study, as a result of examining the simple correlation analysis between the kinematic analysis results and cognitive function, a significant relationship was found in several items. However, it should be noted that these results included the effects of confounding factors. It has been reported that a reduction in the number of teeth and occlusal support can negatively affect both cognitive function and masticatory ability [10,11,23]. Furthermore, patients with dementia have a high prevalence of sarcopenia [24], which is also known to negatively impact masticatory efficiency [12]. To diagnose sarcopenia, measuring grip strength or walking speed, in addition to skeletal muscle mass, is necessary [25]. However, in this study, grip strength measurements were not performed since participants with moderate or severe dementia may not have fully understood the examiner’s instructions and may not have demonstrated their original strength. Since patients requiring severe nursing care and having difficulty getting up from a chair by themselves were included, walking speed was excluded from the evaluation. Therefore, as it was not possible to diagnose sarcopenia, the skeletal muscle index was included as a confounding factor in this study. It has also been reported that the skeletal muscle index is associated with masticatory function [26]. Three confounding factors were set in this study, the Eichner index, ASMI, and MNA-SF, which revealed significant correlations in the simple correlation analysis. Partial correlation analysis showed that cognitive function was significantly correlated with the number of circular motions and circular motion frequency, even after adjusting for these factors. These results suggest that, as cognitive function declines, mandibular movements during mastication decrease in circular motion and increase in linear motion.

When examining the association with each domain of ABC-DS, the masticatory movement changes were associated with ADL function and cognitive function but not with BPSD. Additionally, the circular motion frequency was clearly decreased in the severe group compared to the other groups, while no significant difference was found between the moderate and mild groups. This suggests that residents with severe cognitive impairment have clearly altered mandibular movements during mastication. The brain regions that control masticatory movements have been elucidated in many animal studies. The facial area of the precentral cortex in the cerebrum directly controls the movements of the jaw and tongue during mastication, playing a crucial role in the formation of masticatory patterns [27]. Cortical mastication plays a significant role in smoothly progressing the series of movements from placing food into the mouth, forming a bolus, and transitioning to the swallowing movement [28]. Rhythmic opening and closing movements are considered to be controlled by a central pattern generator (CPG) located in the brainstem [29]. Although this study did not examine the participants’ brains, it is possible that the changes in masticatory movement may have been caused by the decreased function of several brain regions related to mastication.

Previous research also revealed that people with dementia are at risk of choking [30], since they eat in dangerous ways, such as eating rapidly and compulsively (eating everything in front of them without stopping) and/or consuming large bolus sizes [31]. Another possible cause is that care recipients may ingest food that is not suitable for their masticatory function [32]. As a decline in masticatory function is difficult to detect visually, caregivers may not realize that a dietary pattern is unsuitable for residents until a mishap occurs. For residents with moderate to advanced dementia, caregivers should regularly check the movement of the lower jaw while chewing. If there is little circular motion, choking accidents may be avoided by changing to a diet that allows for the easy formation of a bolus.

This study had some limitations. First, this study was conducted in two general nursing care facilities in Japan, but it did not take into account the influence of regional or facility characteristics. Second, only one type of test food was used, and it was unclear whether it could be consistent when using other foods. When chewing the rice cracker used in this study, no effort is required to crush it, but the more it is crushed, the finer it breaks into pieces, so dexterous movement is required to put the pieces together to form a bolus. It is possible that these characteristics of the test food affected the masticatory movement. Additionally, many more participants in this study were excluded than expected, resulting in a small sample size. As many participants had moderate or severe cognitive decline, they could not understand our instructions and were not patient. For example, there were some cases where the participant stopped chewing, started talking, and made large facial movements. In such cases, the images were retaken up to two times, but many participants still had to be excluded because images suitable for analysis could not be taken. As a result, selective bias occurred. Finally, as this study was conducted as a cross-sectional study, differences in the participants’ skeletal structure or other individual factors might have influenced the results. Therefore, it is necessary to continue conducting follow-up surveys in the future. Furthermore, we plan to conduct studies using different test foods or measurement methods to further elucidate the masticatory movements of patients with dementia.

## 5. Conclusions

This study analyzed the mandibular movements during mastication in nursing home residents. The circular motion frequency significantly decreased as cognitive function declined. This may make it more difficult for residents with cognitive decline to ingest normal solid foods. Caregivers need to take this into account to ensure that residents with dementia can safely continue to take oral intake.

## Figures and Tables

**Table 1:** Characteristics.

Age (years)	88.1 [6.1]
male/female	15/48 (24/76)
GCDI (1/2/3/4/5)	9/8/19/20/7 (14/13/30/32/11)
Cerebrovascular disease	20 (32)
BMI (kg/m^2^)	21.03 [3.05]
MNA-SF (normal/at risk/malnutrition)	8/48/7 (13/76/11)
ASMI (kg/m^2^)	6.12 [1.30]
CC (cm)	21.0 [3.1]
Dietary form (normal/soft/minced)	32/17/14 (51/27/22)
ABC-DS mild/moderate/severe	77.0 [19.8] 21/18/24 (33/29/38)
Eichner index (A1/A2/A3/B1/B2/B3)	47/0/1/4/9/2 (75/0/2/6/14/3)

Presented as mean [standard deviation] or number (%). GCDI; Government-Certified Disability Index, BMI; body mass index, MNA-SF; short-form mini-nutritional assessment, ASMI; appendicular skeletal muscle index, CC; calf circumference, ABC-DS; ABC dementia scale.

**Table 2:** Correlation coefficient with kinematic results.

	Masticatory Time	Number of Cycles	Cycle Frequency	Total Change Amount	Speed		Number of Cycle Motions		Circular Motion Frequency
					Average	Maximum	Circular	Linear	
Age	0.055	0.054	0.048	0.111	0.031	0.138	0.022	0.019	−0.027
Cerebrovascular disease (+; 1)	0.085	0.138	–0.004	0.248	0.199	0.159	0.116	0.063	0.039
GCDI	0.012	−0.112	−0.131	−0.067	−0.151	−0.130	−0.197	−0.060	−0.036
BMI	−0.001	−0.055	−0.224	−0.023	0.069	0.117	0.048	−0.166	0.197
MNA-SF	0.204	0.168	−0.119	0.104	0.043	0.016	0.371 **	−0.156	0.325 **
ASMI	−0.108	0.036	0.119	−0.062	0.008	−0.079	0.105	−0.090	0.132
CC	−0.091	0.015	−0.010	−0.056	0.096	0.125	0.016	−0.011	0.062
Dietary form	0.075	−0.007	−0.108	0.189	−0.004	0.021	−0.206	0.227	−0.233
ABC-DS	−0.051	0.132	0.250 *	−0.062	0.194	0.021	0.500 **	−0.405 **	0.601 **
Domain A	0.023	0.066	0.164	0.068	0.202	0.183	0.432 **	−0.324 **	0.526 **
Domain B	0.096	0.048	−0.082	−0.089	−0.098	−0.107	0.118	−0.205	0.231
Domain C	0.022	0.184	0.217	0.003	0.182	0.023	0.497 **	−0.304 *	0.504 **
Eichner index	0.123	0.149	0.044	0.042	−0.092	−0.141	0.227	−0.031	0.065

Spearman’s rank correlation coefficient; *p* < 0.05: *, *p* < 0.01: **. GCDI: Government-Certified Disability Index, BMI: body mass index, MNA-SF: short-form mini-nutritional assessment, ASMI; appendicular skeletal muscle index, CC; calf circumference, ABC-DS; ABC dementia scale.

**Table 3:** Partial correlation coefficient adjusted for MNA-SF, ASMI, and Eichner index.

ABC-DS		Masticatory Time	Number of Cycles	Cycle Frequency	Total Change Amount	Speed		Number of Cycle Motions		Circular Motion Frequency
						Average	Maximum	Circular	Linear	
Total score	r	–0.152	0.063	0.220	–0.133	0.094	0.027	0.409 *	–0.357 *	0.616 **
	*p*	0.248	0.631	0.091	0.311	0.475	0.837	0.001	0.005	<0.001
Domain A	r	–0.089	0.042	0.112	0.007	0.094	0.157	0.363 *	–0.339 *	0.473 **
	*p*	0.498	0.748	0.392	0.958	0.473	0.232	0.004	0.008	<0.001
Domain B	r	–0.025	0.013	0.038	–0.053	–0.031	–0.065	0.051	–0.034	0.155
	*p*	0.851	0.923	0.776	0.689	0.817	0.622	0.699	0.798	0.237
Domain C	r	–0.051	0.092	0.163	–0.074	0.070	0.008	0.367 *	–0.265 *	0.472 **
	*p*	0.699	0.484	0.213	0.577	0.598	0.954	0.004	0.040	<0.001

*p* < 0.05: *, *p* < 0.01: **, MNA-SF: short-form mini-nutritional assessment, ASMI; appendicular skeletal muscle index, ABC-DS; ABC dementia scale.

## Data Availability

The data that support the findings of this study are available on request from the corresponding author. The data are not publicly available due to privacy or ethical restrictions.
